# Supplementary material for: Machine Learning Accelerates Crystallization for Structure Determination
Source: Angew Chem Int Ed Engl. 2026 May 4;65(25):e1218503. doi: 10.1002/anie.1218503 (PMC13266958; doi:10.1002/anie.1218503)

# checkCIF/PLATON report

Structure factors have been supplied for datablock(s) lcz104

THIS REPORT IS FOR GUIDANCE ONLY. IF USED AS PART OF A REVIEW PROCEDURE FOR PUBLICATION, IT SHOULD NOT REPLACE THE EXPERTISE OF AN EXPERIENCED CRYSTALLOGRAPHIC REFEREE.

No syntax errors found. CIF dictionary Interpreting this report

# Datablock: lcz104

---

| | | | |
|---|---|---|---|
| Bond precision: | C-C = 0.0102 A | | Wavelength=1.54184 |
| Cell: | a=9.9293(3) | b=37.1724(8) | c=10.6654(3) |
| | alpha=90 | beta=99.098(2) | gamma=90 |
| Temperature: | 100 K | | |

| | Calculated | Reported |
|---|---|---|
| Volume | 3887.03(18) | 3887.03(18) |
| Space group | P 21/c | P 1 21/c 1 |
| Hall group | -P 2ybc | -P 2ybc |
| Moiety formula | C30 H25 Ag3 F18 N6 O5 | C15 H3 Ag3 F18 N6, C15 H22 O5 |
| Sum formula | C30 H25 Ag3 F18 N6 O5 | C30 H25 Ag3 F18 N6 O5 |
| Mr | 1215.17 | 1215.17 |
| Dx,g cm-3 | 2.076 | 2.076 |
| Z | 4 | 4 |
| Mu (mm-1) | 13.258 | 13.258 |
| F000 | 2360.0 | 2360.0 |
| F000’ | 2371.10 | |
| h,k,lmax | 12,47,13 | 12,46,13 |
| Nref | 8288 | 7971 |
| Tmin,Tmax | 0.195,0.233 | 0.480,1.000 |
| Tmin’ | 0.125 | |

Correction method= # Reported T Limits: Tmin=0.480 Tmax=1.000
AbsCorr = MULTI-SCAN

Data completeness= 0.962 Theta(max)= 77.780

R(reflections)= 0.0593( 6971) wR2(reflections)= 0.1597( 7971)

S = 1.070 Npar= 563

---

The following ALERTS were generated. Each ALERT has the format
**test-name_ALERT_alert-type_alert-level.**
Click on the hyperlinks for more details of the test.

---

## Alert level C

```
PLAT042_ALERT_1_C Calc. and Reported MoietyFormula Strings  Differ     Please Check
              Calc: C30 H25 Ag3 F18 N6 O5
              Rep.: C15 H3 Ag3 F18 N6, C15 H22 O5
PLAT213_ALERT_2_C Atom F01Q            has ADP max/min Ratio .....        3.4 prolat
PLAT220_ALERT_2_C NonSolvent   Resd 1  F   Ueq(max)/Ueq(min) Range        3.3 Ratio
PLAT342_ALERT_3_C Low Bond Precision on  C-C Bonds ...............    0.01016 Ang.
PLAT906_ALERT_3_C Large K Value in the Analysis of Variance ......      5.295 Check
PLAT911_ALERT_3_C Missing FCF Refl Between Thmin & STh/L=    0.600         11 Report
                1  0  0,   0  4  0,  -1  2  1,   0  2  1,  -2  1  2,   3 15  3,
                3 17  3,   4 23  3,  -3 34  7,  -3 34  8,  -2 25 10,
PLAT971_ALERT_2_C Check Calcd Resid. Dens.  0.94Ang From Ag01           1.62 eA-3
```

---

## Alert level G

```
PLAT003_ALERT_2_G Number of Uiso or U(i,j)  Restrained non-H-Atoms          4 Report
PLAT004_ALERT_5_G Polymeric Structure Found with Maximum Dimension          1 Info
PLAT007_ALERT_5_G Number of Unrefined Donor-H Atoms .............           1 Report
              H005
PLAT083_ALERT_2_G SHELXL Second Parameter in WGHT  Unusually Large      27.47 Why ?
PLAT186_ALERT_4_G The CIF-Embedded .res File Contains ISOR Records          2 Report
PLAT242_ALERT_2_G Low    'MainMol' Ueq as Compared to Neighbors of       C013 Check
PLAT242_ALERT_2_G Low    'MainMol' Ueq as Compared to Neighbors of       C019 Check
PLAT242_ALERT_2_G Low    'MainMol' Ueq as Compared to Neighbors of       C01K Check
PLAT242_ALERT_2_G Low    'MainMol' Ueq as Compared to Neighbors of       C01M Check
PLAT242_ALERT_2_G Low    'MainMol' Ueq as Compared to Neighbors of       C01N Check
PLAT242_ALERT_2_G Low    'MainMol' Ueq as Compared to Neighbors of       C01O Check
PLAT333_ALERT_2_G Large Aver C6-Ring C-C Dist C00W     -C012      .      1.44 Ang.
PLAT335_ALERT_2_G Check Large C6 Ring C-C Range C00W     -C012           0.17 Ang.
PLAT720_ALERT_4_G Number of Unusual/Non-Standard Labels ..........         87 Note
              Ag01    Ag02    Ag03    F004    O005    H005    O006    O007
              F008    F009    F00A    F00B    N00C    F00D    F00E    F00F
              N00G    N00H    N00I    F00J    N00K    F00L    F00M    N00N
              F00O    O00P    C00Q    H00A    H00B    C00R    F00S    F00T
              O00U    C00V    C00W    F00X    C00Y    H00C    H00D    C00Z
              H00Z    C010    C011    H01A    H01B    C012    C013    C014
              H014    C015    C016    H01C    H01E    F017    C018    C019
              C01A    H01F    H01G    H01H    C01B    C01C    C01D    H01D
              C01E    C01F    H01I    H01J    C01G    C01H    H01K    H01L
              C01I    H01M    H01N    H01O    F01J    C01K    C01L    C01M
              C01N    C01O    C01P    H01P    H01Q    H01R    F01Q
PLAT860_ALERT_3_G Number of Least-Squares Restraints .............         24 Note
PLAT910_ALERT_3_G Missing FCF Reflection(s) Below Theta(Min)[Deg]=       4.36 Note
```

```
              0  2  0,
PLAT912_ALERT_4_G Missing # of FCF Reflections Above STh/L=  0.600        305 Note
PLAT913_ALERT_3_G Missing # of Very Strong Reflections in FCF ....          1 Note
             -2  1  2,
PLAT941_ALERT_3_G Average HKL Measurement Multiplicity ..........        2.5 Low
PLAT969_ALERT_5_G The 'Henn et al.' R-Factor-gap value ..........      3.181 Note
              Predicted wR2: Based on SigI**2  5.02 or SHELX Weight 14.93
PLAT978_ALERT_2_G Number C-C Bonds with Positive Residual Density.          0 Info
```

---

```
   0 ALERT level A = Most likely a serious problem - resolve or explain
   0 ALERT level B = A potentially serious problem, consider carefully
   7 ALERT level C = Check. Ensure it is not caused by an omission or oversight
  21 ALERT level G = General information/check it is not something unexpected

   1 ALERT type 1 CIF construction/syntax error, inconsistent or missing data
  14 ALERT type 2 Indicator that the structure model may be wrong or deficient
   7 ALERT type 3 Indicator that the structure quality may be low
   3 ALERT type 4 Improvement, methodology, query or suggestion
   3 ALERT type 5 Informative message, check
```

---

### Validation response form

Please find below a validation response form (VRF) that can be filled in and pasted into your CIF.

```
# start Validation Reply Form
_vrf_PLAT042_lcz104
;
PROBLEM: Calc. and Reported MoietyFormula Strings  Differ     Please Check
RESPONSE: ...
;
_vrf_PLAT213_lcz104
;
PROBLEM: Atom F01Q            has ADP max/min Ratio .....        3.4 prolat
RESPONSE: ...
;
_vrf_PLAT220_lcz104
;
PROBLEM: NonSolvent   Resd 1  F   Ueq(max)/Ueq(min) Range        3.3 Ratio
RESPONSE: ...
;
_vrf_PLAT342_lcz104
;
PROBLEM: Low Bond Precision on  C-C Bonds ..............    0.01016 Ang.
RESPONSE: ...
;
_vrf_PLAT906_lcz104
;
PROBLEM: Large K Value in the Analysis of Variance ......      5.295 Check
RESPONSE: ...
;
_vrf_PLAT911_lcz104
;
PROBLEM: Missing FCF Refl Between Thmin & STh/L=    0.600         11 Report
RESPONSE: ...
;
```

```
_vrf_PLAT971_lcz104
;
PROBLEM: Check Calcd Resid. Dens.  0.94Ang From Ag01            1.62 eA-3
RESPONSE: ...
;
# end Validation Reply Form
```

It is advisable to attempt to resolve as many as possible of the alerts in all categories. Often the minor alerts point to easily fixed oversights, errors and omissions in your CIF or refinement strategy, so attention to these fine details can be worthwhile. It is up to the individual to critically assess their own results and, if necessary, seek expert advice.

**PLATON version of 17/09/2025; check.def file version of 17/09/2025**

# duplicate check

**No duplication found**

**Datablock lcz104** - ellipsoid plot

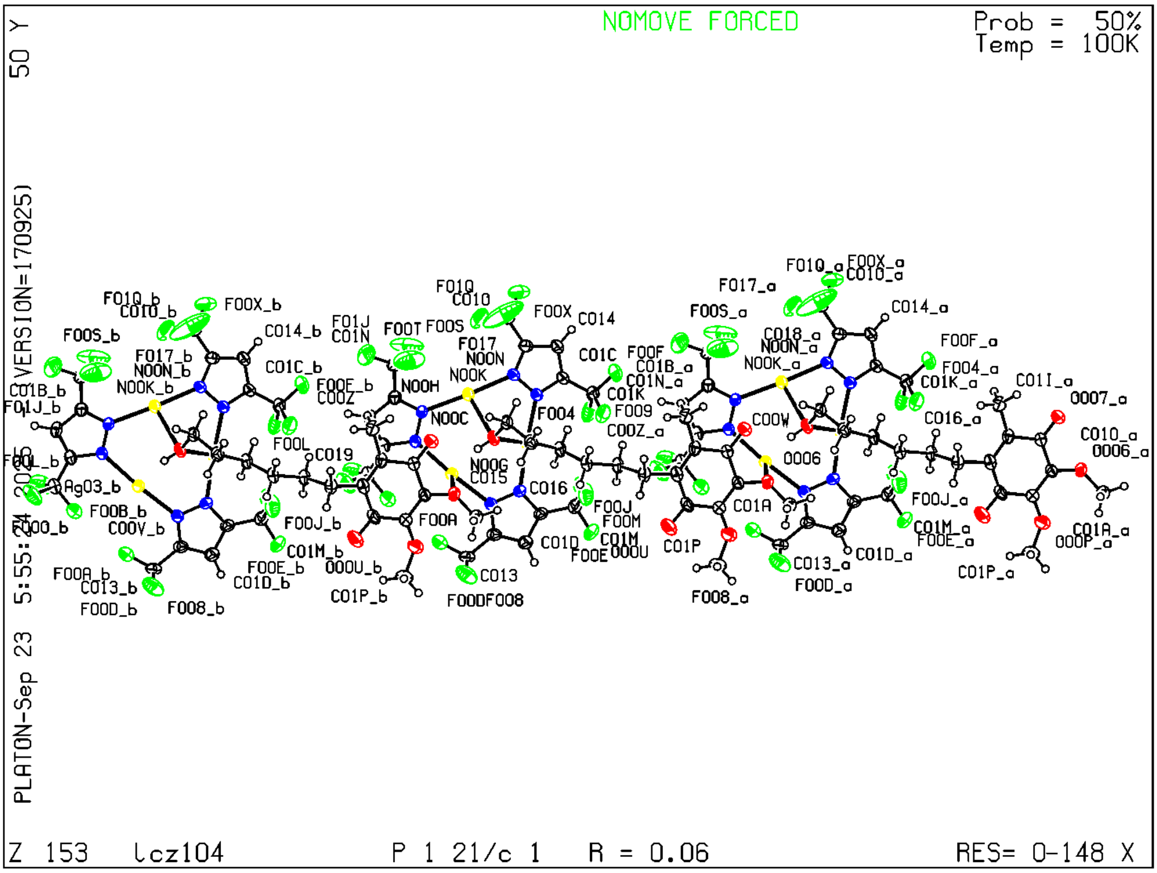

Supplement: Supplementary file 2 — Supporting File 2: anie72462‐sup‐0002‐DataFile.zip. [file ANIE-65-e1218503-s002.zip › supplementary_materials_crystallographic_data submit.zip!/cif_and_checkcif(1-114)/Ag3Pz3_104/Ag3Pz3_104_checkcif.pdf]
